# Optical Oxygen Micro- and Nanosensors for Plant Applications

**DOI:** 10.3390/s120607015

**Published:** 2012-05-25

**Authors:** Cindy Ast, Elmar Schmälzlin, Hans-Gerd Löhmannsröben, Joost T. van Dongen

**Affiliations:** 1 NanoPolyPhotonik, Fraunhofer Institute for Applied Polymer Research, Geiselbergstr. 69, 14476 Potsdam-Golm, Germany; E-Mail: elmar.schmaelzlin@iap.fraunhofer.de; 2 Energy Metabolism Research Group, Max Planck Institute of Molecular Plant Physiology, Am Mühlenberg 1, 14476 Potsdam-Golm, Germany; E-Mail: Dongen@mpimp-golm.mpg.de; 3 Department of Physical Chemistry, Institute of Chemistry, University of Potsdam, Karl-Liebknecht-Str. 24-25, 14476 Potsdam-Golm, Germany; E-Mail: loeh@chem.uni-potsdam.de

**Keywords:** oxygen sensor, biosensors, microsensors, nanosensors, endogenous sensor proteins, dual-frequency phase‐modulation, phosphorescence quenching, plant science

## Abstract

Pioneered by Clark's microelectrode more than half a century ago, there has been substantial interest in developing new, miniaturized optical methods to detect molecular oxygen inside cells. While extensively used for animal tissue measurements, applications of intracellular optical oxygen biosensors are still scarce in plant science. A critical aspect is the strong autofluorescence of the green plant tissue that interferes with optical signals of commonly used oxygen probes. A recently developed dual-frequency phase modulation technique can overcome this limitation, offering new perspectives for plant research. This review gives an overview on the latest optical sensing techniques and methods based on phosphorescence quenching in diverse tissues and discusses the potential pitfalls for applications in plants. The most promising oxygen sensitive probes are reviewed plus different oxygen sensing structures ranging from micro-optodes to soluble nanoparticles. Moreover, the applicability of using heterologously expressed oxygen binding proteins and fluorescent proteins to determine changes in the cellular oxygen concentration are discussed as potential non-invasive cellular oxygen reporters.

## 1. Introduction

Molecular oxygen (O_2_; here often simply referred to as “oxygen”) is an essential factor for most life forms on Earth and its vital role in respiratory energy metabolism has been well described. When the availability of oxygen for respiration is reduced (hypoxia) or depleted (anoxia), severe consequences for cell viability arise. Hypoxic conditions can occur when the resupply of oxygen to a cell does not keep pace with its oxygen consumption. This is for example a typical phenomenon in tumors due to the extremely high respiration rate of cancer cells. While the oxygen distribution is widely studied in animals and mammalian tissues, it is much less well characterized in plant cells. Plants are sessile organisms and therefore depend on their direct environment for the supply of substrate and nutrients. The availability of oxygen in the atmosphere as an essential substrate for plant metabolism is usually underestimated for plants, because of their autogenous photosynthetic oxygen production. However, during the night plants are not photosynthetically active and also non-green tissues such as roots rely on the supply of oxygen from the environment. Additionally, plants lack an active distribution system for oxygen and depend on diffusion of oxygen through the tissue. When the rate of oxygen consumption exceeds the resupply of oxygen from the environment, the plant internal oxygen concentration can drop well below ambient. Indeed, the internal oxygen concentration of cells in the center of plant tissue can be about five times less than the concentration of cells at the surface [1,2]. In addition to these steep internal oxygen concentration gradients, external oxygen deficiency or depletion is regularly occurring in the rooting area after rain or flooding, when oxygen diffusion is hampered due to water-filled spaces in the soil. The lack of oxygen can damage the root system, leading to repressed growth and development of the whole plant. However, with oxygen being a key metabolite, oxygen depletion induces metabolic as well as morphological rearrangements [3], adaptive processes and protective responses that are essential to understand because of their impact on plant cultivation and crop production [4]. Plant productivity could be greatly improved, if the endogenous hypoxia could be circumvented or reduced [5,6].

The development of oxygen sensors has greatly contributed to our knowledge on the oxygen distribution in plant tissue and the regulation of oxygen consumption by plant cells. Still, measuring oxygen concentrations in plant tissue encompasses several plant specific technical problems that do not occur when oxygen is measured in animal cells. Therefore, this review discusses the state of the art of optical oxygen sensor methods with a special emphasis on the problems of their application in plants.

## 2. Polarographic Electrode Sensors *versus* Optical Oxygen Sensors

At present, two categories of methods to assess the oxygen distribution inside tissues are being distinguished, namely electrochemical and optical systems. Much of the early work on oxygen sensing employed Clark-type polarographic electrode sensors, which detect a current flow caused by the chemical reduction of oxygen [7]. Such sensors have been miniaturized to reduce the invasive character of the electrode. High resolution profiles of the oxygen distribution in plant roots were elegantly obtained [8] and ultra-micro electrodes were fabricated to use on single algal cells or purified chloroplasts with a size of 20 and 5 μm respectively [9]. However, microelectrodes display limitations over optically based sensors. They are invasive and consume oxygen, which can cause experimental errors especially when measuring oxygen concentrations in very small volumes such as in a living cell. Nevertheless, they are still more widely used than optical sensors and dominate the current literature of oxygen measurements in plants.

Phosphorescence quenching-based optical oxygen sensing methods have the capability to overcome the limitations of polarographic measurements. The main advantage of optical probes is the potential of extreme miniaturization down to a molecular scale. Furthermore optical sensors are suitable for detecting both dissolved and gaseous oxygen. This review aims to stepwise guide the reader through this subject, by explaining the principle of the measurement technique, introducing the most popular oxygen-sensitive optical probes, new measurement modalities and highlighting particular biological applications. Some techniques are established for mammalian cells and tissue only, but provide high potential for plant application and are thus being discussed as well. Furthermore, we will introduce a class of cell-internal/intrinsic proteins as potential oxygen sensors. Endogenous oxygen-binding proteins such as plant hemoglobin or fluorescent proteins have shown to exhibit oxygen sensitive properties. They bear the great advantage of being genetically encoded and therefore can be expressed inside cells to directly report on the cellular oxygen concentration without additional chemical treatment and physical damage of the cells. Oxygen sensing proteins would thus be of particular value for fundamental physiological research.

## 3. Fundamentals of Oxygen Sensing by Photoluminescence Quenching

**Figure 1.**
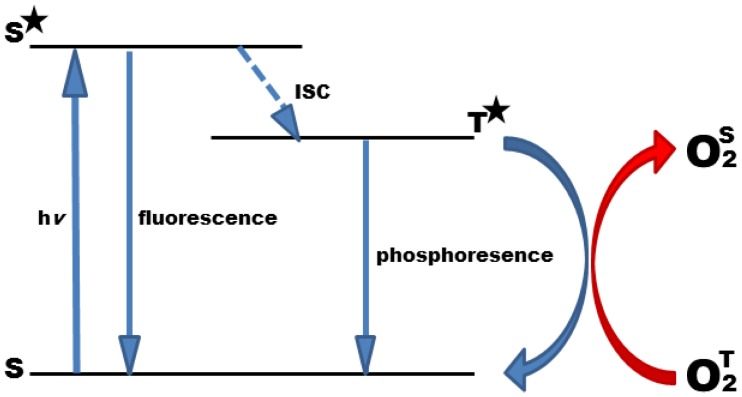
Jablonski Diagram describing the possible states of the indicator molecule, *i.e.*, a luminophore in the ground state (S) and after absorption of radiation (hv) to higher energetic electronic states, namely excited singlet (S*) and excited triplet states (T*). To return to the ground state, the excited molecule emits light of short lived (fluorescence) or long lived emission (phosphorescence), the latter involving a change in the electron spin, a radiationless process termed intersystem crossing (ISC). Molecules in the triplet state are prone to interact with other molecules, like oxygen, and during the process of collisional quenching energy is transferred to the oxygen molecule (O_2_^T^), resulting in singlet oxygen (O_2_^S^).

Oxygen is a quencher of molecular luminescence in general, and photoluminescence quenching is a convenient method to detect oxygen [10,11]. The phenomenon of photoluminescence quenching is described as a dynamic process of the collisional interaction of an analyte molecule with the indicator molecule in its lowest excited electronic state. After collision, energy is transmitted to the analyte resulting in its transfer from the ground state to its excited state, and at the same time in a radiationless decay of the indicator molecule to the ground state (Figure 1). Due to its biradical electronic nature, molecular oxygen has, exceptionally, a triplet ground state. Therefore, triplet-triplet energy transfer is extremely efficient, rendering oxygen detection highly specific with minor cross-reactions, only.

Collisional quenching reduces the luminescence intensity (*I*) and lifetime (τ) of the indicator molecule in a concentration dependent manner and can thus be employed to determine the concentration of the analyte. The quenching behavior can be described by the Stern-Volmer equation:

$$\frac{\tau_{0}}{\tau }=\frac{I_{0}}{I}=1+k_{\text{Q}}\tau_{0}\left[{\text{O}}_{2}\right]$$

where τ_0_ and *I*_0_ are the excited-state lifetime and luminescence intensity in the absence of oxygen, and τ and *I* in the presence of oxygen, respectively. [O_2_] is the partial pressure and concentration of oxygen and *k*_Q_ the bimolecular quenching constant. The latter is dependent on the physico-chemical properties of the system, e.g., solvent parameters, temperature, steric factors, *etc.* [11]. The product *k*_Q_·τ_0_ is also named Stern-Volmer constant. Due to microheterogeneities in the case of solid optical oxygen probes, multiple quenching sites can be involved, leading to non-linear behavior [12]. Therefore, in practice, the calibration curves can be described by a slightly modified Stern-Volmer equation, assuming that only a certain fraction *f* of the indicator molecules is quenched by oxygen [13]:

$$\frac{\tau_{0}}{\tau }=\frac{I_{0}}{I}={\left(\frac{f}{1+k_{\text{Q}}\cdot \tau_{0}\cdot \left[{\text{O}}_{2}\right]}+\left(1-f\right)\right)}^{-1}$$

In contrast to *I*_0_ and *I*, the luminescent lifetimes, τ_0_ and τ, are widely independent of the concentration of the luminophore, and thus are the parameter of choice to measure oxygen concentrations. This holds particularly in cellular systems where absolute dye concentrations are hard to control. It has to be taken into account that the quenching process involves the occurrence of singlet oxygen as by–product, a reactive oxygen species that could damage the biological sample if not protected properly against it [14].

## 4. Oxygen-Sensitive Systems

This section will introduce the most commonly used luminescent oxygen dyes and will discuss their applicability in respect to lifetime, quenching efficiency, spectral properties and stability towards photobleaching, as well as modifications regarding biological application to improve the performance of the sensor.

### 4.1. Oxygen Indicator Dyes

**Figure 2.**
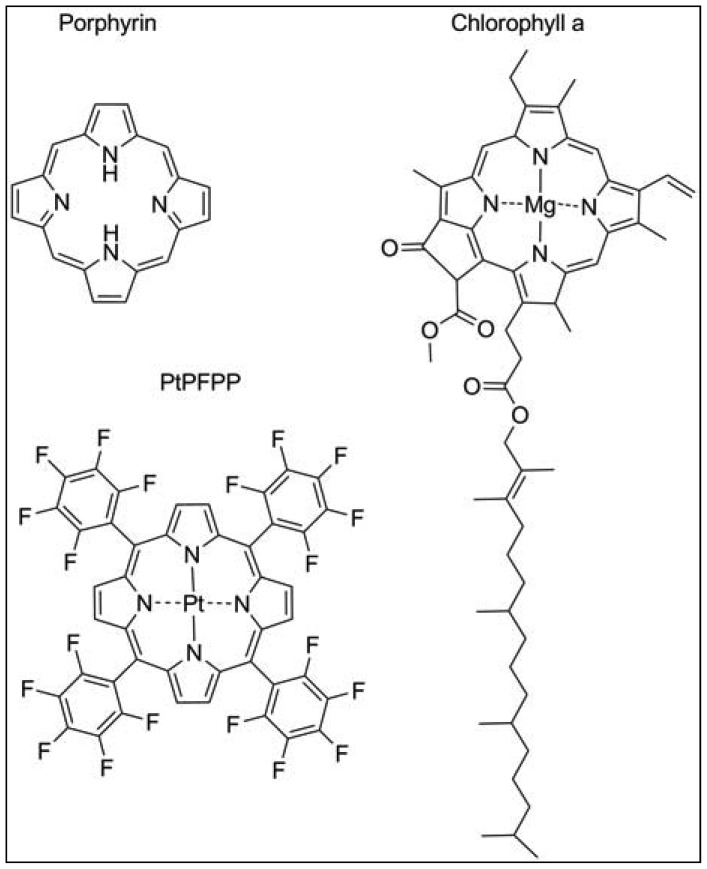
Structural similarities of porphyrin, chlorophyll a and the oxygen probe PtPFPP.

The most widely used exogenous dye structures belong to the organometallic material group. Due to their strong luminescence and long lifetimes, they are highly suitable for the development of oxygen nanosensors. This group is further categorized into metalloporphyrins, among which platinum(II)- and palladium(II)-porphyrins are the most important members and the transition metal complexes with pyridine derivatives, where ruthenium(II) and iridium(III) are worthwhile mentioning. The porphyrin dyes and their metallocomplexes have been investigated for long since oxygen transport in blood, but also photosynthesis is related to naturally occurring porphyrin structures such as heme in hemoglobin or chlorin in chlorophyll (Figure 2) [15]. The spectral properties of oxygen sensors on the basis of *meso*-substituted porphyrin dyes differ according to the metal ion, the chemical structures of the complexing agents and to the ambient matrix. They exhibit distinct absorption spectra with an intense Soret band around 400 nm in the UV and further maxima in the visible range of 500–550 nm. Significant is a bright emission between 630–700 nm which is well preserved at room temperature and in aqueous solutions. The phosphorescence lifetime of these dyes in absence of oxygen is in the range of 40–100 μs for Pt-porphyrins and 400–1,000 μs for Pd-porphyrins. While Pt-porphyrins are well suited for the ambient oxygen range reaching from 0–200 μM, Pd-porphyrins might be the material of choice for an oxygen range below 50 μM [14,16]. Especially the Pt-complex of tetrapentafluorophenylporphyrins (PtPFPP) has to be named, as it is highly suitable for encapsulation in polystyrene micro- and nanobeads. PtPFPP shows high photostability and is water-insoluble which prevents leaching into the biological sample. The PtPFPP structure is illustrated in Figure 2. Application of PtPFPP-doped nanobeads into cells of the algae *Chara corallina* was successfully demonstrated [17]. Recently developed related structures are the Pt- and Pd-complexes of benzoporphyrins which are red-light excitable and possess strong phosphorescence in the near-infrared (NIR) spectral range [18]. These probes would be potentially suitable for measurements in biological systems, because an emission in the NIR range is less interfered by scattering and background fluorescence arising from cell substances. Furthermore, excitation with red light largely prevents from photochemical side reactions within the tissue or the culture medium. Another advantage of emission in the NIR range is that biological tissue is in general relatively transparent in the range 800–1000 nm so that the penetration depth of the optical signal might be larger.

From the group of transition metal complexes, Ru(II)-bipyridyl complexes have also been applied in biological oxygen sensing [19]. Excitation occurs in the visible spectral range between 450–460 nm and the emission maximum is found around 610 nm. They exhibit moderate photostability and a high luminescence quantum yield, but compared to the porphyrins their lifetimes are significantly shorter (∼1 μs), which results in a lower sensitivity to oxygen [20]. Depending on the concentration range to be detected and the imaging technique available, this can also be advantageous, since shorter lifetimes lead to a faster image acquisition. Chemical stability and phototoxicity seems to be the limitation of these dyes. While porphyrin dyes rarely show phototoxicity effects, there is evidence that selected Ru(II) complexes do [21].

Ir(III)-based polypyridyl (ppy) complexes show intense green luminescence at 512 nm, but excitation ranges into the UV-part of the spectrum at around 375 nm, which is inconvenient for biological samples [22]. Substitution of the ppy-ligands with ppy-NPh_2_ leads to a red shift of the emission (520 and 560 nm) with excitation at 355 nm. Depending on the solvent used, it displays high sensitivity towards oxygen [23]. Cyclometalated coumarin complexes of Ir(III) instead are excited at 444 and 472 nm respectively, and emit at around 563 nm. They have long lifetimes of 11 μs and a high luminescence quantum yield but lack photostability under continuous performance, limiting their application [24]. Recently developed Ir(III)-porphyrin complexes extend the spectrum of oxygen sensitive materials. They show strong phosphorescence at room temperature and high quantum yields, as described for the Pt(II)- and Pd(II)-porphyrins. However, their chemical structure is not planar. Axial ligands were introduced, that can influence water-solubility and covalent coupling to biomolecules, but their potential for biological applications still has to be presented [25].

In summary, phosphorescent Pt(II)- and Pd(II)-porphyrins dominate the current research field on oxygen sensing due to their high signal intensity and photostability, as well as their low cytotoxicity.

### 4.2. Probe Format

Usually, the indicator dye is encapsulated within an oxygen-permeable matrix. Immobilized sensor dyes show strongly increased signal intensities and are shielded against undesirable interference. Important properties of the matrix are chemical and photochemical stability, rigidity, oxygen permeability, transparency, processability and negligible swelling in liquids. Established matrices are polystyrene, polymethylmethacrylate, fluoropolymers and glass-like materials formed by sol-gel processes [11,26,27]. Depending on the intended use, the sensor material is finished to various probe configurations, e.g., blocks, films, powders, coatings or micro- and nanospheres, which will be described in this chapter. In the case of water-soluble oxygen probes, macromolecules are substituted for a solid matrix [28,29].

#### 4.2.1. Solid State and Fiber Optic Sensors

Solid state sensors involve the immobilization of the sensor dye to a support or device, such as a membrane, optical fiber or microplate. To create thin-film sensors, foils containing the luminescent probe or surfaces of glass, like slides, plates or whole containers are casted with a mix of the dye solution and polymer encapsulation matrix [30]. They can be employed by growing the tissue directly on the film to analyze oxygen gradients over a longer period of time [31,32]. Very detailed data for root respiration was obtained by growing a whole plant in a container, where the inner walls were covered with the PtPFPP-sensor foil and oxygen concentrations were monitored for two weeks [33]. These types of planar sensor films allow measurements of larger samples, bacterial cultures or whole organs and simultaneous screening of microplate wells. However, the method is inconvenient for the use of single plant cells, which is why oxygen optical microsensors, also referred to as micro-optodes, enjoy a greater popularity in the field of single cell application.

Micro-optodes consist of a tapered glass fiber, the tip usually of a diameter of 10–50 μm and coated with the sensor material. Kopelman and Rosenzweig were greatly involved in the fabrication a sub-micron optical fiber sensors and designed the first pulled oxygen fiber sensor, published in 1995 [34]. The sensing mix consisting of polyacrylamide and a ruthenium complex as dye was attached to the tip that was preincubated with a photopolymerization reagent and the fiber was coupled to a light source, like a laser [35]. Nowadays, these fibers combine the two features of excitation source and transmission of the phosphorescence signal to the detector, to limit the set-up to one fiber only. For data acquisition, oxygen-dependent phosphorescence lifetime is determined by a phase modulation technique. However, the conventional technique applied for commercially available measurement instruments using micro-optodes is hampered by the interference of the fluorescence of the biological sample, in particular the chlorophyll autofluorescence of plant tissue. A dual-frequency modulation technique was developed, which takes advantage of the longer lifetime or the phosphorescent oxygen probe in comparison to the fluorescence of the chlorophyll. This technique will later be discussed in further detail [17]. Micro-optodes are versatile devices that can be applied for extra- and intracellular measurements of oxygen, independently of the cell's origin being plant or mammalian tissue [36,37].

In plants, a custom-made micro-optode, with a tip diameter of less than 20 μm was successfully applied to measure the oxygen concentration in living root nodules of *Lotus japonicus*. This fiber employed the PtPFPP-dye as oxygen sensitive dye, whose phosphorescent signal normally interferes with the plant autofluorescence but can be overcome by applying the two-frequency phase modulation technique. One major disadvantage of optodes is the disruption of the tissue by introducing the glass fiber into the tissue, which not only damages and stresses the cell, but may also allow oxygen to enter from the outside. These negative side effects can be strongly reduced by using ultra-thin glass fibers [38]. Yet, the manufacturing of such delicate fibers is time consuming, which is why different sensor formats are desirable.

#### 4.2.2. Soluble Sensors

The term soluble sensor is used for sensor particles that are not attached to a surface, but can freely diffuse or move through biological fluids. In general, this can be achieved through linkage of the probe to different molecules or polymers. Active development took place to improve solubility, chemical stability and cell-impermeability to facilitate extracellular measurements of oxygen. However, increasing demand for intracellular applications calls for cell-loading techniques that are minimally invasive and least stressful for the cells. Delivery techniques, like microinjection were improved, but also self-loading probes were introduced and are already widely established for mammalian cells and tissues, also showing potential for plant application.

In solutions, Pt(II)- and Pd(II)-porphyrins are moderately quenched by oxygen. However, their hydrophobic character and tendency to aggregate is disadvantageous for using them as water-soluble sensors. This can be somewhat avoided by conjugating the dyes to hydrophilic molecules, such as the serum protein albumin. They remain highly phosphorescent and sensitive to oxygen, but are better soluble and coagulate less in solution. Because this complexation is due to hydrophobic interactions only, limitations like the migration of the dye to other proteins and a general variable sensor composition has to be taken into account [39].

These limitations are partly overcome by the commercially available complex MitoXpress [40] that comprises of an oxygen sensitive dye conjugated to a macromolecular carrier. It is now widely used to determine external oxygen concentrations of isolated mitochondria, cells and environmental samples. Limits of this probe are slow and cell-specific cell-loading and the need for a transfection agent for the probe uptake if applied intracellularly [14].

Another approach to confer the oxygen probe water-soluble is via encapsulation with dendritic polyglutamic chains, which create diffusion barriers and therefore regulate the sensitivity for oxygen. Additionally, the periphery is modified with PEG-residues, to enhance solubility, which also helps to prevent interactions of the probe with the biological environment. A family of dendritic oxygen probes was described with excitation bands spanning the entire UV–VIS–NIR spectrum [29]. Linkage to proteins or PEG usually leads to higher sensitivity towards oxygen and shielding from interferences, but involves a dramatic increase in size, which can be a drawback for cell entry. Conjugation to smaller molecules, like cell-penetrating peptides (CPPs) is described for intracellular targeting and oxygen sensing. The class of CPPs consists of short, positively charged peptides that are able to translocate across cell membranes, carrying a cargo of a much higher mass and size [41]. Dyes, mainly Ru(II)-complexes and Pt(II)-coproporphyrins, were linked to short polyarginine or proline-rich peptides with a length of 8–10 amino acids and are passively transported across the plasma membrane of mammalian cells where they are retained [20,42–44]. The mechanism of entry and transport is not fully understood, but most likely, the uptake is facilitated via endocytosis [45]. Without the need of an additional endocytosis activating transfection agent, as described for MitoXpress, CPPs offer a convenient and rapid cell self-loading capability.

Currently, the described modifications of sensor material have only been applied in mammalian systems. However, only recently it was discovered, that CPP translocation is also possible for plant cells [46], opening new perspectives for plant research using these short peptides as nanocarriers. The cell wall of plant cells is a major feature that distinguishes them from animal cells. Protoplasts presently offer the best comparison to mammalian cells and so far, tobacco protoplasts [46], but also tomato and onion root cells were tested with CPPs, like the proteins and peptides transactivator of transcription (TAT) [47], transportan and arginine-rich intracellular domain (AID) [48]. Nevertheless, literature on CPPs escorting derivatives of metalloporphyrins or other described oxygen sensor structures into plant cells is still limited but they bear a high potential for future investigations.

#### 4.2.3. Micro- and Nanoparticle Sensors

Probes encapsulated in micro- or nanoparticles combine the positive features of solid-state dyes with those of soluble probes. Particulate sensors have a protective shell that reduces interactions, retains stability, and prevents interference with other proteins as well as leakage of the dye into the cell [49]. At the same time they are soluble, minimally invasive and can be applied in living systems by various delivery techniques like pico-injection, gene gun or liposomal transfer [50]. The most prominent candidates of this category are the nanosensors named PEBBLEs (Probes Encapsulated By Biologically Localized Embedding), originally developed by Kopelman and colleagues and applied on macrophages, for intracellular studies. These were fabricated for different analytes, including oxygen [51].

Polymers like polyacrylamide and polystyrene were tested for biocompatibility and permeability towards oxygen and delivery techniques into mammalian cells were discussed [50,52]. PEBBLEs have proven to be suitable for applications inside mammalian cells, though the red phosphorescence signal would interfere with the autofluorescence of the plant chlorophyll if applied in plant cells. Therefore, a microbead-based plant and algae-applied system employing the PtPFPP probe was developed, successfully circumventing the interference of the plant autofluorescence by using the two-frequency phase modulation technique described later. The probe is encapsulated in microbeads of polystyrene, which barely interferes with biological systems. PtPFPP has the advantage of being completely insoluble in water, preventing leaching into the sample. The beads however do not possess self-loading abilities and need to be loaded into the cell via microinjection, which of course may lead to minor injuries, but does not necessarily affect the cell viability [17].

Besides CPP-mediated translocation, only invasive techniques are available to address intracellular delivery of the oxygen sensing probes and with respect to plant application, there has been a lot more research described regarding microelectrodes. Yet, outstanding brightness and photostability, simple fabrication and long-term storage, due to the possibility of sterilization are great advantages of micro- and nanoparticles oxygen sensors. The only limitation of microbeads is the size, which ranges from 40‐300 nm per particle and can lead to cell damage especially if long-term studies as desired [14]. For the latter case, endogenous proteinaceous sensors would be the perfect choice, like they are already developed for important analytes and metabolites such as calcium, sucrose and hormones [53–55].

#### 4.2.4. Endogenous Sensor Proteins

The ideal oxygen nanosensor to measure intracellular oxygen concentrations should be non-invasive and least perturbing for the biological system it is applied on. Genetic modification of living cells to express and produce the luminescent sensor structures themselves would suit these demands perfectly, circumventing the need of microinjection of a foreign compound.

Plants, like mammals, possess heme-containing oxygen binding proteins that are ubiquitously existent throughout the whole plant kingdom and are homologous with vertebrate myo- and hemoglobins [56]. Plant symbiotic hemoglobin, also termed leghemoglobin binds molecular oxygen within the nodules of legume plants, thereby reducing the free oxygen concentration in the plant tissue surrounding the bacteria that live symbiotically within the nodules. Extensive studies demonstrate the applicability of leghemoglobin to be used as a cell-internal oxygen sensor dye [36]. Measurements of the respiration rate and oxygen permeability of legume nodules were performed by spectrophotometric means. The ratio of transmittance of red and infra-red light was calculated and used as value for oxygenation. Leghemoglobins like other heme-containing structures possess a porphyrin moiety (Figure 2), whose emission in the red but not in the infrared spectral range is oxygen-dependent. Oxygenation of leghemoglobin after application of varying external oxygen concentrations was reliably detected in a non-invasive manner [36]. However, nodules are an exceptional plant tissue, and no other example is known in which the heme-protein accumulates to similar high concentrations as in nodules. Nevertheless, *in vitro* analysis showed a high oxygen affinity due to a low oxygen dissociation constant for the non-symbiotic hemoglobins, making this protein a promising candidate to be used as intracellular oxygen-sensing dye. There is some recent evidence for plant hemoglobins being involved in NO detoxification [57]. Therefore, in order to create a reliable nanosensor for oxygen, cross-sensitivities of other compounds need to be further examined, and possibly taken into account.

In the context of oxygen-binding respiratory proteins, hemocyanins as non-plant oxygen-binding proteins from arthropodes need to be mentioned. These proteins contain copper instead of iron in their active site, which gets oxidized upon oxygen binding and can be monitored spectroscopically because of an absorbance around 340 nm (molar extinction coefficient ε = 20,000 M^−1^ cm^−1^). Additionally, quenching of the intrinsic tryptophan fluorescence is linearly related to the bound oxygen in the oxygenated protein, due to FRET from the excited tryptophan to the oxygen-bound active site [58]. Encapsulated in a silica gel matrix, hemocyanins were used to study oxygen binding curves [59].

Very recently, the first Foerster resonance energy transfer (FRET)-based sensor for observing intracellular oxygen concentrations in the bacterium *E.coli* was described. This fluorescent protein-based biosensor for oxygen, termed FluBO is genetically encoded and takes advantage of the sensitivity of fluorescent proteins towards oxygen. It combines the yellow fluorescent protein (YFP) that requires oxygen for the maturation of the chromophore and the hypoxia-tolerant flavin-binding fluorescent protein (FbFP) through a short linker. In this biosensor, FRET can only occur in the presence of oxygen, but not in its absence, therefore monitoring low oxygen conditions [60].

There are more interesting reports in the literature where fluorescent proteins are used as intrinsic indicators of low oxygen levels. For the green fluorescent protein (GFP), an oxygen-dependent red shift in fluorescence upon irradiation with blue light was reported. A low oxygen environment and a short exposure to blue light are sufficient to generate a stable red fluorescent form of the GFP protein. This phenomenon, described with the term anaerobic redding, is only observed at low oxygen concentrations of 0–2%, and is reversible after re-oxygenation, resulting in a fast disappearance of the red fluorescent form. So far, *in vivo* photoconversion was demonstrated using various GFP mutants, localized in different cellular compartments and organisms like *E. coli* [61] and fission yeast [62] and mammalian cell lines such as human epithelial kidney cells [63] and COS-7 cells, but not yet in plants. The human hepatoma cell line Hep3b expressing mitochondria-tagged GFP successfully demonstrated this oxidative redding process to visualize oxygen gradients in cell monolayers [64]. Also cyan variants of fluorescent proteins are capable of this photoconversion. This ability however seems to depend on the chromophore structure, because only the proteins with a tyrosine-based chromophore display conversion to the red state under anaerobic conditions [63]. For plant application it has to be considered, that the red autofluorescence of the chlorophyll of green plant cells might interfere with the red signal from GFP after anaerobic redding.

In summary, the family of fluorescent proteins bears a huge potential as intracellular oxygen nanosensors, since they can be expressed in any organism and are available in various colors within the visible spectral range [65].

## 5. Optical Measurement Modalities and Systems

The oxygen probes introduced in this review are based on luminescence quenching. Therefore, detection of the oxygen distribution can be performed either by intensity or lifetime measurements. Since signal intensities are prone to concentration changes, ratiometric referencing with pairs of oxygen-sensitive and insensitive dyes were developed. Since the polymer matrix traps the dye non-covalently into internal pores, a second luminophore that is not quenched by oxygen can be easily added to serve as a reference dye [52].

Instead, detection of the probe's lifetime is widely independent of the concentration, and particularly the long lifetimes of the phosphorescent Pt(II)- and Pd(II)-porphyrin dyes are technically convenient for lifetime-based systems. As lifetime measurements are often based on phase modulation, the focus of this topic is on a recently developed dual-frequency phase modulation technique [17,37,38], which overcomes the disadvantages of the autofluorescence of biological tissues, particularly of plants. Among imaging systems, based on intensity or lifetime detection, are time-resolved fluorometry (TR–F), life cell imaging, laser-scanning microscopy, fluorescence and phosphorescence lifetime imaging (FLIM). These methods have recently been reviewed thoroughly [14], and are outside the scope of this paper.

### Dual-Frequency Phase Modulation Technique

A straightforward approach is to excite the sensor with a pulse of light and to record the emitted signal with appropriate temporal resolution. Even some commercial instruments employ this principle of measurement, but it is often unsatisfying for real-time measurements. Due to the microheterogeneity of a solid state sensor, the resulting decay curves are stretched exponentially [12]. The analysis needs computing time, and a correlation of the evaluated parameters with oxygen concentration is not trivial. Furthermore, the pulse repetition rate is limited for long-lifetime phosphorescence, which especially extends the measurement time for weak signals.

Therefore, commercial instruments for optical oxygen measurements mostly use phase modulation, in which the sensor is excited with sinusoidally modulated light. Depending on the decay time, the emitted phosphorescence signal is temporally delayed, which results in a phase shift between the excitation and phosphorescence light. The corresponding oxygen concentration is calculated subsequently using a calibration curve. A drawback of the standard phase modulation technique is that the phase shift is strongly interfered by background fluorescence in the spectral range of the sensor emission. In the case of green plant tissue, the red fluorescence of chlorophyll superimposes the sensor signal and reduces the detected phase shifts. Therefore, for real-time monitoring of oxygen within plant tissue, a special two-frequency phase modulation technique was developed, which masks fluorescence. The technique is based on the fact that the time delays of all background signals can be assumed to be negligible compared to the microseconds lifetime of the oxygen sensor's phosphorescence. Measuring the respective phase shifts at two different modulation frequencies simultaneously allows quantification and subsequently mathematical removal of all background signals, which are in-phase with the excitation light [17]. In brief, this two-frequency modulation method works with two sinus generators that provide sinus signals with different frequencies. The sinus signals are electronically superposed. The waveform generated in this way is used to amplitude-modulate the excitation light. The detected sensor signal is analyzed by a lock-in amplifier unit, which separates the two frequencies and measures the respective phase shifts in reference to the excitation signal. With the help of the apparent phase shifts, the fraction of in-phase background signals can be calculated [17]. Not only fluorescence is eliminated, but also residual excitation light, which may unintentionally reach the detector. The latter is especially advantageous for extremely miniaturized probes, were the excitation intensity is several orders of magnitudes higher than the probe signal. Even though the evaluated decay time is an average value, generated by sensor dye molecules embedded in a microheterogeneous probe, the correlation with oxygen concentration is unambiguously possible.

## 6. Current Plant Applications and Future Prospects

There is substantial interest in obtaining detailed information on the oxygen distribution in or outside cells, in particular with respect to plant applications. The creation of two- and three-dimensional oxygen maps is a new and convenient tool to demonstrate oxygen profiles.

Based on the results obtained from the measurements using micro-optodes, oxygen maps of seeds were established, to show tissue oxygen levels and changes in response to environmental factors. Several crop plant species, ranging from maize to wheat were investigated, demonstrating the applicability of the technique. The micro-optode was stepwise inserted into the seed and local oxygen measurements were carried out. To prevent diffusion of oxygen into the seed, the entry point had to be sealed. Following the measurement, seeds were dissected to identify the particular zones to set up the corresponding oxygen profile. The benefits from these maps are the identification of regions within the seed, that are susceptible to diffusional impedance of gas exchange, gas diffusion pathways, as well as oxygen release due to photosynthesis. This information is of particular interest for crop growth and development [5].

Very detailed oxygen maps on root respiration of whole plant roots in soil were reported recently, where the inner walls of containers were casted with oxygen sensor foils and in which the plants were grown for two weeks. The oxygen concentration was monitored continuously, allowing the visualization of diverse plant activities, like photosynthesis and metabolic acclimation. Here, additionally the water uptake was mapped by neutron radiography, to measure these two important parameters in parallel. The combined imaging can yield a more profound interpretation of the data, since oxygen distributes differently in the aqueous or gaseous phase. This method should be applicable to naturally growing plants, providing the possibility to non-invasively map oxygen in the soil over long periods of time [33].

Sophisticated *in-silico* three-dimensional models for gas exchange in fruit were established, using microscale geometry only. This approach aims to fill the gaps, where local oxygen measurements are not feasible or practical, in particular for very well-defined positions. The gas exchange is simulated for fruit, incorporating the structure of the fruit tissue by means of synchroton radiation tomography. The obtained data were compared with experimental results of oxygen measurements, thus validating the model but at the same time demonstrating the necessity of having experimental data available [66].

**Figure 3.**
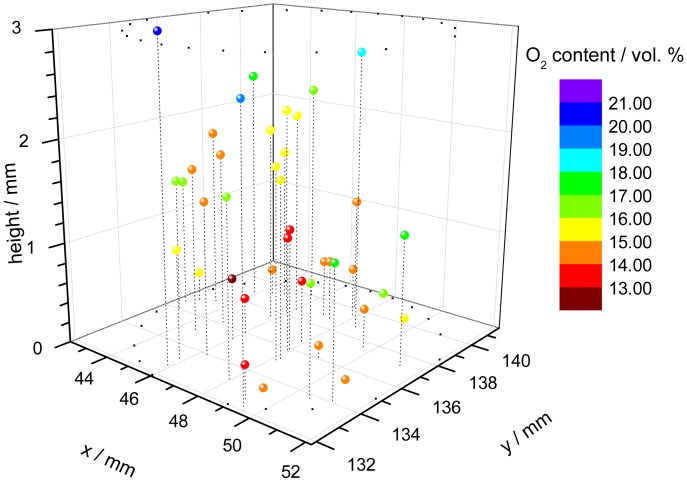
3D-plot of the oxygen concentration in a cell culture of chondrocytes after four days of growth. Every sphere represents one microprobe located within the sample. The colors of the spheres display the measured oxygen concentrations at their positions. The circulary arranged small black dots mark the position of the upright cylindrical cell carrier.

One application of the dual-frequency phase modulation technique is to measure the oxygen distribution within a cell carrier [67]. For this purpose, polymer microspheres, which were stained with the indicator dye, were suspended within a hydrogel cylinder. The cylinder is used as carrier for a culture of cells, in this case chondrocytes because they prefer low oxygen conditions in comparison to e.g., skin cells. The oxygen content is an important parameter to monitor and to control the tissue growth in bioreactors. A confocal microscope was used to focus every single microprobe. The individual decay time of each microbead was measured with a specially equipped microscope, consisting of the electronic hardware unit, external light source and detection optic [68]. Since the spatial position of the single probes is known, a three-dimensional pattern of the oxygen concentration can be assembled (Figure 3).

## 7. Conclusions

Although plants produce molecular oxygen themselves via photosynthesis, many plants tissues have to deal with hypoxic conditions due to the fact that no active oxygen-transport mechanisms exist in plants. This has severe consequences for the plant's respiratory energy metabolism and therefore for plant growth and crop yield. In order to obtain a better understanding of the physiology of low-oxygen stress responses in plants, methods to measure oxygen in plants is of particular importance. A variety of methods—ranging from miniaturized Clark-type electrodes to various optical oxygen sensors—has been developed in the recent years. In particular optical sensing techniques provide novel tools that are expected to enable oxygen measurements at cellular or even sub-cellular level in the near future and are extensively discussed here. Future prospects and aims are to encourage interdisciplinary research on oxygen sensing, to improve the existing techniques and methods and to gain profound knowledge of the effects of oxygen in particular inside plant cells.
